# A Novel Protistan Trait Database Reveals Functional Redundancy and Complementarity in Terrestrial Protists (Amoebozoa and Rhizaria)

**DOI:** 10.1111/1755-0998.70064

**Published:** 2025-10-30

**Authors:** Jule Freudenthal, Martin Schlegel, Michael Bonkowski, Kenneth Dumack

**Affiliations:** ^1^ Terrestrial Ecology, Institute of Zoology, Cluster of Excellence on Plant Sciences (CEPLAS) University of Cologne Köln Germany; ^2^ Aquatic Ecosystem Analyses, Institute for Integrated Natural Sciences University of Koblenz Koblenz Germany; ^3^ Biodiversity and Evolution, Institute of Biology University Leipzig Leipzig Germany; ^4^ German Centre for Integrative Biodiversity Research (iDiv) Halle Jena Leipzig Leipzig Germany

**Keywords:** ecological traits, functional trait analysis, functional trait reference database, microbial ecology, predator–prey, soil

## Abstract

The inclusion of functional traits of protists in environmental sequencing surveys, in addition to the traditional taxonomic framework, is essential for a better understanding of their roles and impacts on ecosystem processes. We provide a database of functional traits for a widespread and important clade of protists—the Amoebozoa—based on extensive literature research in eight trait categories: Habitat, locomotion, nutrition, morphology, morphotype, size, spore formation, and disease‐relatedness. The comparison of community traits of the Amoebozoa with sympatric but highly divergent Cercozoa (Rhizaria) revealed both convergent evolution of morphology or locomotion and distinct differences in habitat preference and feeding selectivity. Amoebozoa seem to be rather unselective in their prey choice compared to Cercozoa. Indeed, the feeding preferences of Amoebozoa appeared to be related to cell size, whereas Cercozoa selectively feed on prey. Applications to metatranscriptomic data from soil, litter, and bark surfaces revealed differences in the average community trait compositions and ecosystem functioning, such as an increased proportion of disease‐related Amoebozoa in soil or different proportions of nutrition types of Amoebozoa and Cercozoa on bark. This database will facilitate ecological analyses of sequencing data and improve our understanding of the diversity of adaptations of Amoebozoa to the environment and their functional roles in ecosystems.

## 1 Introduction

Refined information on the functional diversity of organisms, in addition to the traditional taxonomic framework, may greatly improve our knowledge of their function in ecosystem processes (Bouskill et al. 2012; Krause et al. 2014) but also, for example, how abiotic and biotic drivers shape communities (Briones 2014; Fiore‐Donno et al. 2019). To meet the analytical demands of environmental sequencing projects, trait‐based data must be collated and tools developed to easily assign functional traits to existing sequencing databases.

Trait‐based community analyses aim to link species diversity to ecosystem functioning (Lavorel and Garnier 2002; Violle et al. 2007). Traits, on the one hand, determine the performance and fitness of an organism (response traits) by directly reflecting its adaptations to physical, chemical, and biotic environmental drivers. On the other hand, traits such as feeding mode capture their potential impact (effect traits) on the environment and, thus, species' contributions to ecosystem functioning (Krause et al. 2014; Suding et al. 2008). Accordingly, trait‐based community analyses may provide detailed information on the niche space occupied by communities (Lennon et al. 2012) or covered by a taxonomic group (Díaz et al. 2016) but also allow for an upscaling of ecosystem processes (Mulder et al. 2013).

Trait‐based surveys are widely established for plants, animals, and prokaryotes (Beier et al. 2022; Bouskill et al. 2012; Louca et al. 2016). However, a sound functional understanding of the super‐diverse communities of microbial eukaryotes is challenging, as their over 20 phyla comprise a multitude of completely independent evolutionary trajectories (Ruggiero et al. 2015). Furthermore, taxonomic and functional diversity are generally not necessarily coupled (Louca et al. 2016). For example, closely related taxa may exhibit different predatory impacts (Glücksman et al. 2010). Accordingly, studies covering a broad range of the diversity of microbial eukaryotes may so far provide only limited information on their functions (Aslani et al. 2022; Giachello et al. 2023; Köninger et al. 2023).

Over the past decade, there has been an increase in trait‐based environmental surveys focusing on the lesser‐investigated part of the microbial diversity: the protists (Amacker et al. 2022; Fiore‐Donno et al. 2019; Flues et al. 2017; Jauss et al. 2021; Lamentowicz et al. 2020). Protists, a paraphyletic assemblage of mostly unicellular eukaryotes, represent the vast majority of microbial eukaryotic diversity. Protists fulfill numerous and diverse ecological functions in both terrestrial and aquatic ecosystems, including primary production, the exertion of distinct predation patterns, and parasitism of plants and animals. To deepen our understanding of their functional roles in ecosystem processes, it is crucial to include functional traits. For example, the metabolic basis of protistan functional traits has been used to identify the main drivers of shifts between net heterotrophy and autotrophy in the oceans and to establish models predicting phytoplankton blooms (Alexander et al. 2015). Moreover, the importance of symbioses among planktonic eukaryotes was only revealed after compiling the planktonic Protist Interaction DAtabase (PIDA, Bjorbækmo et al. 2020). Similarly, in terrestrial ecosystems, the application of functional traits has revealed seasonal increases in consumer pressure that promote more diverse and evenly distributed prey communities, highlighting the role of biotic interactions in shaping microbial food webs over time (Fiore‐Donno et al. 2024).

In soils, Amoebozoa and Cercozoa (Rhizaria) are the most dominant terrestrial protistan supergroups (Domonell et al. 2013; Dumack et al. 2016; Fiore‐Donno et al. 2024; Urich et al. 2008; Voss et al. 2019). A detailed trait database exists for the Cercozoa and Endomyxa (Dumack et al. 2020). This trait database allows an easy assignment of traits to environmental sequences and thus enables functional insights into the structure of microbial food webs. Fiore‐Donno et al. (2019) showed a relative increase in the abundance of shell‐bearing Cercozoa with drier soils, supporting the long‐assumed function of their shells; that is, increased drought resistance due to reduced evaporation.

**FIGURE 1:**
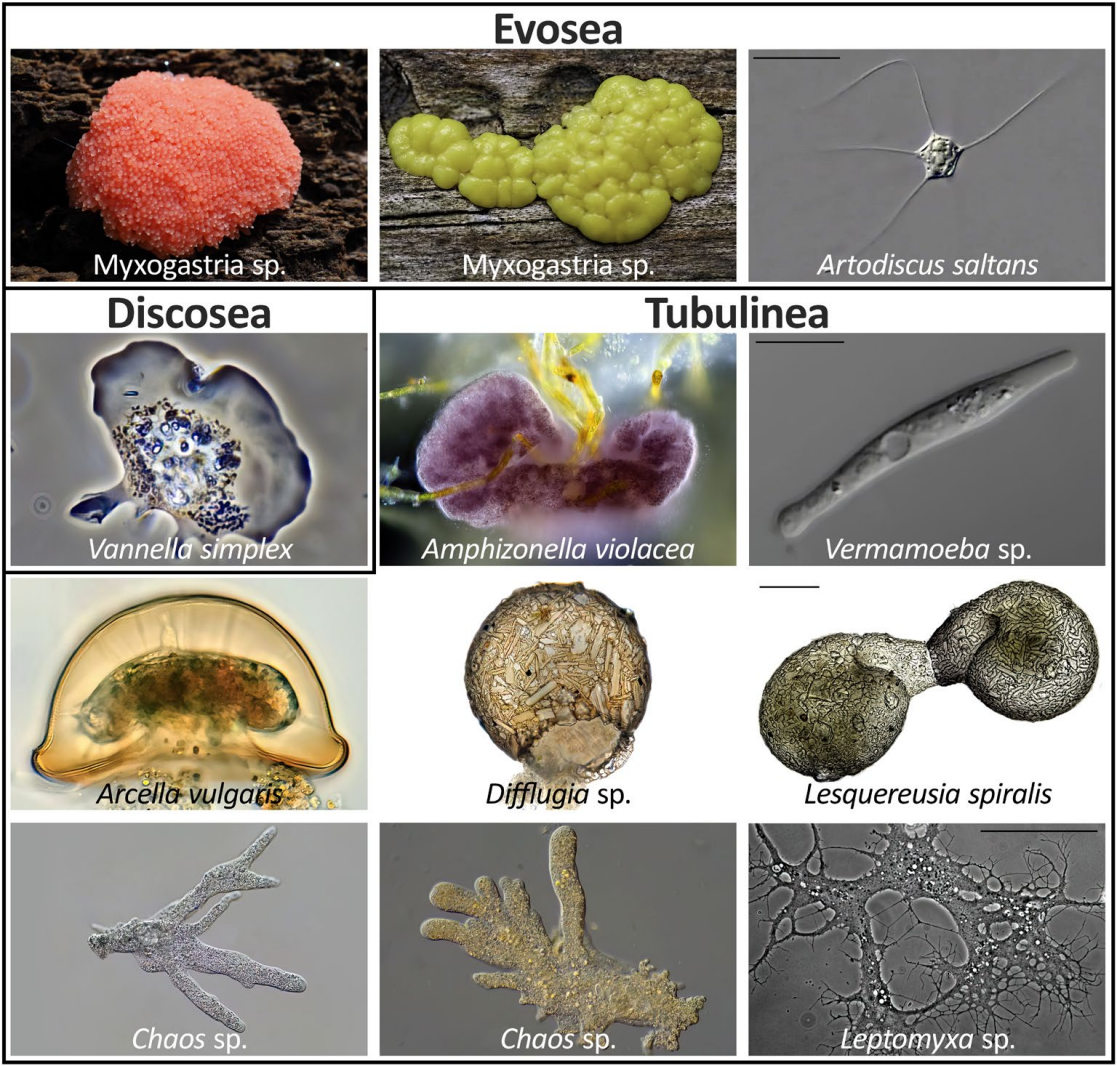
Overview of the morphological diversity of the Amoebozoa. The graphic shows (1) the Evosea, represented by Myxomycetes sp. and *Artodiscus saltans* (Conosea), (2) the Discosea, represented by *Vannella simplex* (Flabellinia), and (3) the Tubulinea, represented by *Amphizonella violacea* (Corycida), *Vermamoeba* sp. (Echinamoebida), *Arcella vulgaris* , *Difflugia* sp., *Lesquereusia spiralis* (Arcellinida), *Chaos* sp. (Euamoebida), and *Leptomyxa* sp. (Leptomyxida).

The supergroup Amoebozoa is equally diverse (Figure 1) and abundant as Cercozoa and comprises three major lineages (Kang et al. 2017; Tekle et al. 2022): First, the Evosea, which include some taxa with flagellated cells and complex life cycles, and others that are giant, such as the plasmodia of Myxomycetes (clades: Cutosea and Conosea). Second, the Discosea, comprising the Flabellinia, characterised by flattened cells with separate hyaloplasm from which (lobose) subpseudopodia protrude (orders: Stygamoebida, Thecamoebida, Dactylopodida, Vannellida, Dermamoebida), and the Centramoebida, some of which possess scales (orders: Acanthamoebida, Pellitida, Himatismenida). Third, the Tubulinea, comprising shell‐bearing but mostly naked lobose amoebae, with highly variable cell sizes ranging from 20 μm to several centimetres; taxa with larger cells often form branching (ramose) or network‐forming (reticulose) pseudopodia (orders: Leptomyxida, Euamoebida, Arcellinida, Echinamoebida). Moreover, the Amoebozoa include important human parasites like *Acanthamoeba* spp., *Balamuthia mandrillaris*, and *Entamoeba histolytica* (Fiore‐Donno et al. 2016; Geisen et al. 2014; Tice et al. 2016; Walochnik 2018). Traditional approaches had early on identified the ecological importance of Amoebozoa in soil systems as part of microbial food webs, shaping community composition as both predators and prey, and facilitating nutrient cycling; for example, mineralisation of nitrogen in the soil that promotes plant growth (Azam et al. 1983; Clarholm 1985; de Ruiter et al. 1995). Unfortunately, the widespread use of “general eukaryotic” primers in metabarcoding studies has led to a consistent and dramatic underrepresentation of amoebozoan sequences in surveys of terrestrial eukaryote diversity (Fiore‐Donno et al. 2016; Geisen, Laros, et al. 2015; Lentendu et al. 2014). In contrast, metatranscriptomics does not suffer from these extreme primer biases and has led to sequencing results largely concordant with microscopic surveys illustrating the dominance of Amoebozoa (Fiore‐Donno et al. 2024; Freudenthal et al. 2022; Heck et al. 2023; Voss et al. 2019). Now that the molecular methodology to assess the taxonomic richness and diversity of Amoebozoa is established, a trait database is highly needed to understand the diversity of their functional roles in terrestrial and aquatic communities.

Here, we provide a trait database for Amoebozoa to serve as a common reference and to facilitate functional ecological studies. We showcase the usage of amoebozoan traits on recently published metatranscriptomic data of soil, leaf litter, and bark surfaces, and we compare the traits of the two most dominant soil protistan supergroups—Amoebozoa and Cercozoa.

## 2 Materials and Methods

### 2.1 Trait Database Design and Justification of Genus‐Level Trait Assignment

As a baseline for our literature research, we screened the curated diversity of amoebozoan 18S rDNA sequences in the PR^2^ database v. 5.0.0 (Guillou et al. 2012), which is based on the taxonomic framework of Burki et al. (2020), to retrieve all currently included amoebozoan genera. As most traits (e.g., nutrition, locomotion, morphotype) in protists are conserved within genera (Dumack et al. 2020). In addition, sequences in reference databases are typically not assigned to species, as short reads in environmental sequencing data often do not allow for reliable taxonomic assignment at the species level. We attributed traits by means of an extensive literature review, including original descriptions, meta‐analyses, and already collated datasets. All consulted references are provided in the database (Table S1) and in the Data S1. Our aim was to select functional traits that enable ecologically meaningful interpretation of community data and to facilitate robust statistical analyses of sequencing data. To this end, we (1) included both response traits, which reflect adaptations to environmental conditions, and effect traits, which indicate potential influence on ecosystem functions (Krause et al. 2014; Suding et al. 2008), (2) aligned the traits as much as possible with those used in the published Cercozoa trait database (Dumack et al. 2020) to facilitate cross‐group comparability, while also incorporating traits specific to Amoebozoa (e.g., spore formation), (3) focused on traits that were consistently present in most descriptions, and (4) divided each trait category into discrete units such that each taxon is assigned only once per category. Despite our attempts to be as exhaustive as possible, this trait database is a strongly simplified representation of the vast functional diversity of Amoebozoa. Due to the necessary sum of compromises to increase its practical application, it may not fulfill the expectations of taxonomists. For instance, the use of discrete trait categories is particularly challenging for Amoebozoa with complex life cycles (Keller et al. 2022; Tice et al. 2016).

Additionally, certain traits, particularly cell size, may differ considerably even within one genus or across different stages (Berney et al. 2015; Kylin 2001). Therefore, instead of recording size as a continuous variable, we assigned a fixed (common) size range to each genus, which, however, needs to be considered with care, as variability can be large. Accordingly, comments and references are given for each genus (Table S1; Data S1).

### 2.2 Functional Traits

We considered the following functional trait categories for Amoebozoa: prey range, rough morphology, and morphotype, locomotion, known habitat preference, animal disease‐relatedness (whether as vector or immediate parasite), presence/absence of spore formation, and size range (see Table 1; Table S1; Data S1). Prey range states were grouped according to bacterivorous, omnivorous (feeding on bacteria and eukaryotes), eukaryvorous (feeding on fungi, microfauna, algae, or other protists), and saprotrophy. We could not assign more precise states (e.g., fungivorous, algivorous) for a lack of information (or contradictory reports, i.e., likely multiple trophic modes) for most taxa. Morphology was mainly specified by the presence/absence of a shell and flagella. As amoebozoan amoebae, although variable, show well‐recognisable shapes (Smirnov and Brown 2004), we further defined simplified morphotypes; that is, disc, tubule, palm, and reticulate. Two main locomotion modes were recognised: substrate‐bound gliding and free swimming. However, amoebae, amoeboflagellates, and flagellates differ not only in their locomotion, but amoeboid cells are surface feeders, whereas prey capture of flagellates likely is much more selective due to their larger handling time. Habitat states distinguished soil and freshwater taxa from marine taxa, given that soil‐inhabiting and freshwater taxa may easily switch habitats, whereas marine taxa are rarely found in soil/freshwater habitats (Singer et al. 2021; Smirnov and Brown 2004). Genera accommodating species from marine and soil/freshwater habitats were considered to have evolved ubiquitous habitat preferences. Spore formation is an important trait to enhance dispersal and survival in heterogeneous and harsh environments (Geisen et al. 2018). As most protists exhibit dispersal limitation locally and regionally, especially in terrestrial and soil systems, spore formation might impact community turnover, biogeography, and historical diversification (Bates et al. 2013; Martiny et al. 2006; Singer et al. 2019). For simplicity, we did not consider different spore formation strategies.

**TABLE 1:** Functional traits of amoebozoa. The table summarizes the functional traits used to characterize Amoebozoa in this study, including each trait's possible states or categories and their ecological relevance. Full trait database and references are provided in Table S1 and Data S1.

Trait categories	States	Ecological meaning/relevance
Nutrition	Omnivorous, Eukaryvorous, Bacterivorous, Autotroph, Saprotrophic, Plant parasite, Not plant parasite, Animal parasite	Nutritional modes influence community assembly (Singer et al. 2021), biotic interactions (Flues et al. 2017; Gao et al. 2019; Glücksman et al. 2010), and ecosystem functions such as nutrient cycling and carbon fluxes (Bonkowski and Clarholm 2012; Briones 2014; Jassey et al. 2022)
Morphology	Naked flagellate, Naked amoeba, Naked amoeboflagellate, Testate flagellate, Testate amoeba, Testate cell silica, Testate cell organic, Endoparasite	Morphology and morphotype reflect adaptations to microhabitats and substrate (Geisen et al. 2018), environmental stress (e.g., desiccation), defence against predators, or specialised feeding strategies (Dumack et al. 2024; Geisen et al. 2018)
Morphotype	Disc, Tubule, Palm, Reticulate, Star	
Size [μm]	Continuous variable	The size of protists is associated to metabolism, growth rate, trophic position, ecological niches, and environmental responses (Brown et al. 2004; Potapov 2022; Woodward et al. 2005)
Size categories [μm]	≤ 100 μm, ≤ 300 μm, > 300 μm	
Locomotion	Gliding, Swimming, Non‐motile	Reflects microhabitat adaptation and resource exploitation strategies (Dumack et al. 2020; Fiore‐Donno et al. 2019)
Habitat	Ubiquitous, Soil and freshwater, Marine, Gut	Habitat preference reflects environmental tolerance and niche adaptation (Burki et al. 2021)
Disease related?	Yes, No	Identifies disease‐related protists, which may pose ecological and economic threats in; for example, aquaculture or may cause severe diseases in humans and animals (El‐Dib 2017; Lisnerová et al. 2025; Tice et al. 2016; Visvesvara et al. 2007)
Spore formation observed?	Yes, No	Spore formation is associated with survival and dispersal potential in variable or harsh environments (Geisen et al. 2018)

Suggestions for updates can be addressed to the corresponding authors. A full list of the trait categories, their states, and ecological relevance is provided in Table 1. All consulted references for the trait database (Table S1) are provided in the Data S1. An R package for the easy assignment of the traits and updated versions of the database are available at Github (https://github.com/JFreude/FunctionalTraitsAmoebozoa) and Zenodo (DOI: https://doi.org/10.5281/zenodo.15091355).

### 2.3 Statistics

The statistical data analyses were conducted in R v. 4.3.1 (R Core Team 2023). The data were visualised with the R packages ggplot2 v. 3.5.1 and ggpubr v. 0.6.0 (Kassambara 2023a; Wickham 2011). For all analyses, we expanded the trait dataset by including trait data for Cercozoa and Endomyxa (Rhizaria) from an existing functional trait database by Dumack et al. (2020). For convenience, we will refer to Cercozoa and Endomyxa as Cercozoa.

To assess the functional redundancy and complementarity between Amoebozoa and Cercozoa, we compared the relative genus richness across trait states within each trait category and visualised the results using a Sankey diagram. Only taxa for which traits could be assigned were considered for the relative genus richness.

To test for size differences associated with nutritional modes, we compared the sizes of bacterivorous, eukaryvorous, and omnivorous taxa for Amoebozoa and Cercozoa, respectively. If only a size range was given for a taxon, the mean size was calculated. Specifications such as “up to” or “larger than” were not considered; taxa with a size of “up to macroscopic” were regarded as 1000 μm in size. The sizes across feeding types were compared using a Kruskal‐Wallis test and Dunn's post hoc test (rstatix v. 0.7.2::kruskal_test and rstatix v. 0.7.2::dunn_test; Kassambara 2023b). Pairwise comparisons were corrected for multiple testing according to Benjamini and Hochberg (1995).

To showcase the usage of the trait database, we visualised the functional trait composition of Amoebozoa and Cercozoa communities across different terrestrial habitats. We used publicly available metatranscriptomic datasets from bark (Freudenthal et al. 2024), litter (Voss et al. 2019), and soil (Fiore‐Donno et al. 2024). For consistency of the sampling conditions across habitats, we only used soil samples that were collected in the summer rather than winter (see Fiore‐Donno et al. 2024), in line with the sampling period of the bark and litter samples. The mean and standard deviation of the relative community composition for each trait category were calculated and visualised in a point diagram for each habitat and community (Amoebozoa and Cercozoa), respectively.

## 3 Results and Discussion

**FIGURE 2:**
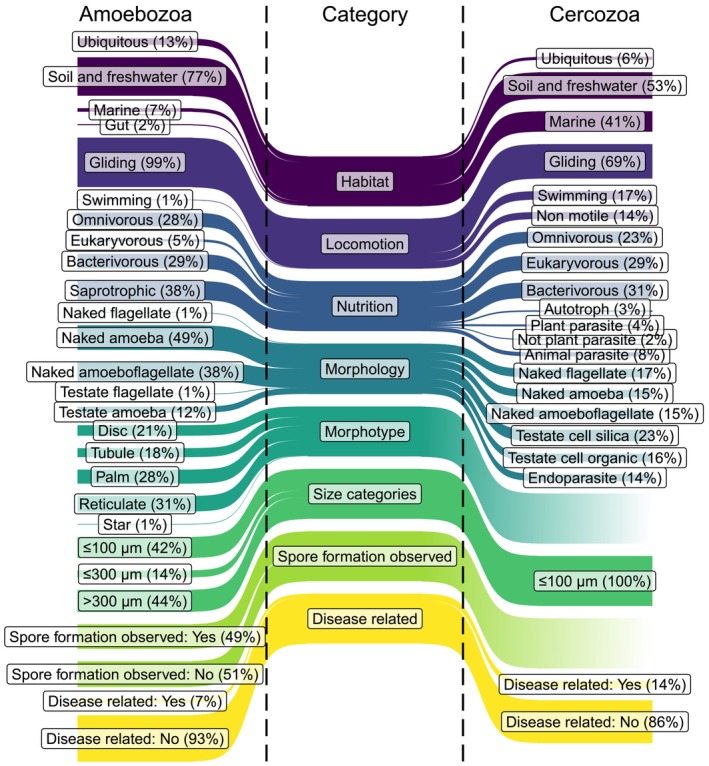
Overview of the relative genus richness per functional trait within each category of the Amoebozoa and Cercozoa databases. The Sankey diagrams show the percentual genus richness calculated for the given traits of each category for the Amoebozoa (left) and Cercozoa (right) databases.

We provide a functional database for Amoebozoa, allowing the easy integration of functional traits into molecular studies, thus facilitating ecological interpretation. The database comprises functional traits; that is, habitat, locomotion, nutrition, morphology, and size (Figure 2; Table 1). Additionally, we included information on morphotype, whether spore formation was observed, and whether they may cause diseases.

A comparison of the genus richness per trait of Amoebozoa with the Cercozoa (Figure 2) revealed both functional redundancy—that is, taxa fulfilling similar ecological roles—and functional complementarity—that is, taxa occupying distinct ecological niches (Barry et al. 2019; Louca et al. 2018). Both groups show similar morphological variation and habitat preferences: They both include shell‐bearing amoebae, naked amoebae, and flagellated taxa and are dominated by gliding species. The latter is assumed to be an adaptation to surface feeding in soil habitats. Moreover, although the majority of both taxa feed on bacteria, either as bacterivores or as omnivores, they also exhibit striking differences, such as the large proportion of eukaryovores in Cercozoa. Further, almost 80% of the known amoebozoan genera occur in soil or freshwater and only a small fraction in marine environments, whereas for Cercozoa, the ratio of soil and freshwater to marine genera is nearly balanced. This habitat‐related difference is attributable to marine‐specialised clades within Cercozoa, such as Chlorarachnea (mostly autotrophic), Phaeodarea (freely swimming taxa with siliceous skeletons), and Ascetosporea (animal parasites). Indeed, the pronounced separation between taxa inhabiting soil/freshwater and marine habitats is observed across all protist groups. Virtually no taxa occur in both habitats, and salinity is considered one of the strongest environmental filters driving this division (Burki et al. 2021; Logares et al. 2009; Singer et al. 2021).

**FIGURE 3:**
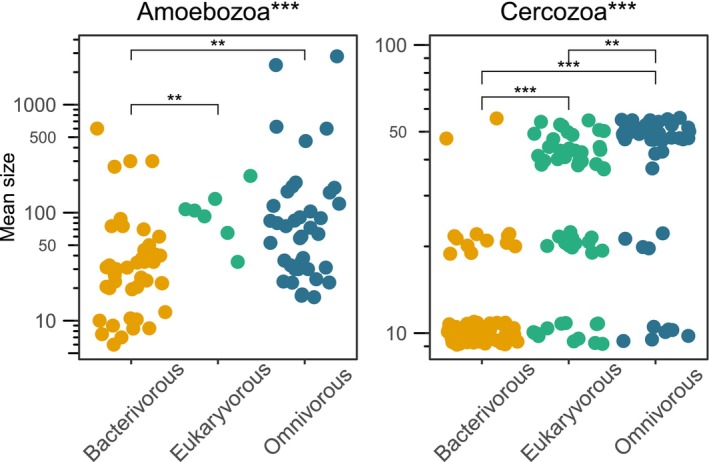
Overview of the association between size and feeding type for Amoebozoa and Cercozoa. The point diagrams show the mean sizes of bacterivorous (yellow), eukaryvorous (green) and omnivorous (blue) for the Amoebozoa (left) and Cercozoa (right) databases. Significant differences across all feeding types (Kruskal‐Wallis test) and of pairwise comparisons of the feeding types (Dunn's test) are indicated with stars in the graph title or the graph, respectively (*p < 0.05; **p < 0.01; ***p < 0.001).

We show that the mean size of Amoebozoa and Cercozoa is associated with their feeding type (Figure 3). Traditionally, most protists were considered to be bacterivorous (Bezemer et al. 2010; de Ruiter et al. 1995). In recent years, however, it has become increasingly clear that many protists indeed exhibit a broad prey spectrum, including algae, fungi, and other heterotrophic protists (Dumack et al. 2019; Estermann et al. 2023; Geisen et al. 2016; Seppey et al. 2017). For testate amoebae, feeding type can generally be inferred from the shell size. Specifically, the aperture size has been shown to constrain the maximum prey size (Fournier et al. 2015; Jassey et al. 2012), although with certain limitations, such as ecological constraints; for example, sensitivity to desiccation in larger‐shelled species (Fournier et al. 2015), or exceptions like highly specialised predators using their shells to attack much larger prey (Dumack et al. 2024). Our comparative analysis of cell size and feeding type across all Amoebozoa and Cercozoa expands this general size–feeding type relationship beyond testate amoebae to all groups of Amoebozoa and Cercozoa. We found that mean cell size significantly differed among feeding types; that is, bacterivorous taxa were significantly smaller compared to eukaryvorous and omnivorous taxa in both Amoebozoa (*p* < 0.001) and Cercozoa (*p* < 0.0001) (see Figure 3). These findings support the utility of cell size as a proxy for feeding type. Although this pattern holds in general, our analyses further indicate a difference within it: While eukaryvorous taxa are relatively common in Cercozoa, they are rare in Amoebozoa, suggesting that smaller Amoebozoa ingest only bacteria, whereas larger ones consume bacteria and single‐celled and multicellular eukaryotes, such as nematodes and fungi (Geisen, Rosengarten, et al. 2015). In other words, if the prey item can be entirely enclosed by an amoebozoan cell, it is suitable prey. Thus, the Amoebozoa are likely much less selective in their prey choice than Cercozoa, but the size of the amoebozoan cell determines which prey can be ingested (Kulishkin et al. 2023). However, few reliable data on feeding preferences exist for Amoebozoa, and more feeding experiments are urgently needed, predominantly in taxa where individual species may differ by several orders of magnitude in cell size, for example, in Variosea (Berney et al. 2015).

**FIGURE 4:**
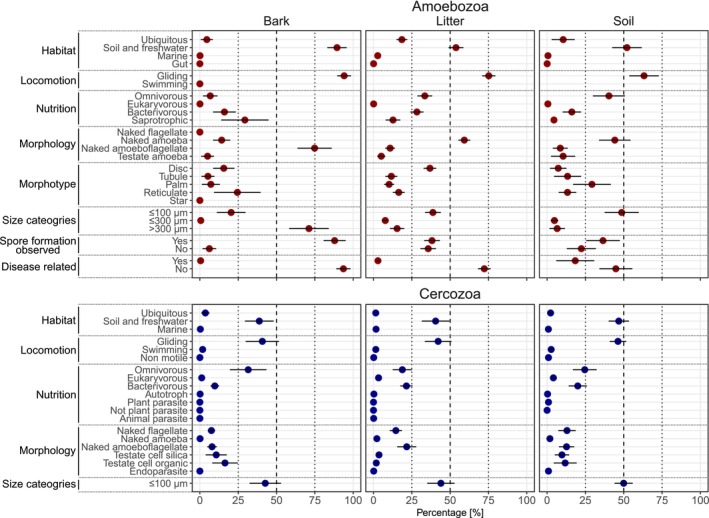
The relative proportion of functional traits of Amoebozoa and Cercozoa communities of bark, litter and soil. The point diagrams show the percentages that were assigned to the given traits of each category for Amoebozoa and Cercozoa communities of bark (*N* = 15), litter (*N* = 18) and soil (*N* = 39), respectively. The points represent the mean and are colour‐coded by Amoebozoa (red) and Cercozoa (blue). The error bars represent the standard deviation. The proportion of taxa with missing trait information is not shown, therefore, percentages within each trait category may not sum to 100%.

We compared environmental metatranscriptomic datasets from soil, leaf litter, and bark surfaces and compared the variation in functional diversity across habitats (Figure 4; Figure S1). Our analyses revealed distinct functional trait composition across habitats, particularly within Amoebozoa. Bark‐associated amoebozoan communities exhibited a unique functional community composition, characterized by a high proportion of reads assigned to saprotrophic taxa (~30%), naked amoeboflagellate morphotypes (~75%), large cell size (> 300 μm, ~70%), and spore‐forming taxa (~90%). These traits reflect the dominance of Myxogastria, which are well adapted to the harsh environmental conditions on bark surfaces through; for example, resistant spore stages (Ing 1994; Shadwick et al. 2009). In contrast, disease‐related Amoebozoa were abundant in soil (~18%) but nearly absent from bark (< 1%), consistent with previous reports that soils harbor a high proportion of disease‐related taxa (Fiore‐Donno et al. 2016; Geisen et al. 2014; Voss et al. 2019). Together, these findings suggest that habitat filtering strongly shapes amoebozoan communities. In contrast, Cercozoa communities were more functionally homogeneous across habitats. This may reflect the dominance of generalist taxa with high dispersal capacity (Finlay 2002; Bahram et al. 2016; Jauss et al. 2020) or indicate functional redundancy, where multiple taxa fulfill similar ecological roles, thus buffering Cercozoa communities against environmental variability (Louca et al. 2018). Furthermore, our analyses showed a large hidden functional diversity (Figure S1), particularly among Cercozoa due to insufficient taxonomic assignment, highlighting the need for future taxonomic classification and species descriptions.

The newly provided trait database for Amoebozoa enables an easy assignment of traits to environmental sequencing surveys. This will allow detecting trade‐offs and evolutionary trajectories in adaptations among different supergroups in protists and deepen our knowledge of the functional diversity of Amoebozoa and their impact on ecosystem functioning.

## Author Contributions

K.D., M.S., and M.B. designed the study. K.D. and J.F. compiled the trait database. J.F. created all figures, performed the statistical analyses, and wrote the R package. J.F. outlined the manuscript; all co‐authors commented on the manuscript and approved the submitted version.

## Disclosure

All contributions to the research are described in the article, in the Supporting Information, and in the Acknowledgments. The benefits of this research accrue from sharing the results and data on public databases.

## Conflicts of Interest

The authors declare no conflicts of interest.

## Supporting information


**Data S1.** men70064‐sup‐0001‐DataS1.docx.

**Table S1.** men70064‐sup‐0002‐TableS1.txt.

## Data Availability

The trait database and an R package for the automatic assignment of the traits to a taxonomy table will be publicly accessible upon publication at GitHub (https://github.com/JFreude/FunctionalTraitsAmoebozoa) and Zenodo (DOI: https://doi.org/10.5281/zenodo.15091355).

## References

[men70064-bib-0001] Alexander, H. , M. Rouco , S. T. Haley , S. T. Wilson , D. M. Karl , and S. T. Dyhrman . 2015. “Functional Group‐Specific Traits Drive Phytoplankton Dynamics in the Oligotrophic Ocean.” Proceedings of the National Academy of Sciences 112: E5972–E5979. 10.1073/pnas.1518165112. PMC4640801 26460011

[men70064-bib-0002] Amacker, N. , Z. Gao , J. Hu , A. L. C. Jousset , G. A. Kowalchuk , and S. Geisen . 2022. “Protist Feeding Patterns and Growth Rate Are Related to Their Predatory Impacts on Soil Bacterial Communities.” FEMS Microbiology Ecology 98: fiac057. 10.1093/femsec/fiac057. 35524686 PMC9126823

[men70064-bib-0003] Aslani, F. , S. Geisen , D. Ning , L. Tedersoo , and M. Bahram . 2022. “Towards Revealing the Global Diversity and Community Assembly of Soil Eukaryotes.” Ecology Letters 25: 65–76. 10.1111/ele.13904. 34697894

[men70064-bib-0004] Azam, F. , T. Fenchel , J. Field , J. Gray , L. Meyer‐Reil , and F. Thingstad . 1983. “The Ecological Role of Water‐Column Microbes in the Sea.” Marine Ecology Progress Series 10: 257–263. 10.3354/meps010257.

[men70064-bib-0086] Bahram, M. , P. Kohout , S. Anslan , H. Harend , K. Abarenkov , and L. Tedersoo . 2016. “Stochastic Distribution of Small Soil Eukaryotes Resulting from High Dispersal and Drift in a Local Environment.” ISME Journal 10: 885–896. 10.1038/ismej.2015.164. 26394006 PMC4796928

[men70064-bib-0005] Barry, K. E. , L. Mommer , J. van Ruijven , et al. 2019. “The Future of Complementarity: Disentangling Causes From Consequences.” Trends in Ecology & Evolution 34: 167–180. 10.1016/j.tree.2018.10.013. 30527960

[men70064-bib-0006] Bates, S. T. , J. C. Clemente , G. E. Flores , et al. 2013. “Global Biogeography of Highly Diverse Protistan Communities in Soil.” ISME Journal 7: 652–659. 10.1038/ismej.2012.147. 23235291 PMC3578557

[men70064-bib-0007] Beier, S. , J. Werner , T. Bouvier , N. Mouquet , and C. Violle . 2022. “Trait‐Trait Relationships and Tradeoffs Vary With Genome Size in Prokaryotes.” Frontiers in Microbiology 13: 985216. 10.3389/fmicb.2022.985216. 36338105 PMC9634001

[men70064-bib-0008] Benjamini, Y. , and Y. Hochberg . 1995. “Controlling the False Discovery Rate: A Practical and Powerful Approach to Multiple Testing.” Journal of the Royal Statistical Society 57: 289–300. 10.1111/j.2517-6161.1995.tb02031.x.

[men70064-bib-0009] Berney, C. , S. Geisen , J. Van Wichelen , et al. 2015. “Expansion of the ‘Reticulosphere’: Diversity of Novel Branching and Network‐Forming Amoebae Helps to Define Variosea (Amoebozoa).” Protist 166: 271–295. 10.1016/j.protis.2015.04.001. 25965302

[men70064-bib-0010] Bezemer, T. M. , M. T. Fountain , J. M. Barea , et al. 2010. “Divergent Composition but Similar Function of Soil Food Webs of Individual Plants: Plant Species and Community Effects.” Ecology 91: 3027–3036. 10.1890/09-2198.1. 21058562

[men70064-bib-0011] Bjorbækmo, M. F. M. , A. Evenstad , L. L. Røsæg , A. K. Krabberød , and R. Logares . 2020. “The Planktonic Protist Interactome: Where Do We Stand After a Century of Research?” ISME Journal 14: 544–559. 10.1038/s41396-019-0542-5. 31685936 PMC6976576

[men70064-bib-0012] Bonkowski, M. , and M. Clarholm . 2012. “Stimulation of Plant Growth Through Interactions of Bacteria and Protozoa: Testing the Auxiliary Microbial Loop Hypothesis.” Acta Protozoologica 51: 237–247. 10.4467/16890027AP.12.019.0765.

[men70064-bib-0013] Bouskill, N. J. , D. Eveillard , D. Chien , A. Jayakumar , and B. B. Ward . 2012. “Environmental Factors Determining Ammonia‐Oxidizing Organism Distribution and Diversity in Marine Environments.” Environmental Microbiology 14: 714–729. 10.1111/j.1462-2920.2011.02623.x. 22050634

[men70064-bib-0014] Briones, M. J. I. 2014. “Soil Fauna and Soil Functions: A Jigsaw Puzzle.” Frontiers in Environmental Science 2: 7. 10.3389/fenvs.2014.00007.

[men70064-bib-0015] Brown, J. H. , J. F. Gillooly , A. P. Allen , V. M. Savage , and G. B. West . 2004. “Toward a Metabolic Theory of Ecology.” Ecology 85: 1771–1789. 10.1890/03-9000.

[men70064-bib-0016] Burki, F. , A. J. Roger , M. W. Brown , and A. G. B. Simpson . 2020. “The New Tree of Eukaryotes.” Trends in Ecology & Evolution 35: 43–55. 10.1016/j.tree.2019.08.008. 31606140

[men70064-bib-0017] Burki, F. , M. M. Sandin , and M. Jamy . 2021. “Diversity and Ecology of Protists Revealed by Metabarcoding.” Current Biology 31: R1267–R1280. 10.1016/j.cub.2021.07.066. 34637739

[men70064-bib-0018] Clarholm, M. 1985. “Interactions of Bacteria, Protozoa and Plants Leading to Mineralization of Soil Nitrogen.” Soil Biology and Biochemistry 17: 181–187. 10.1016/0038-0717(85)90113-0.

[men70064-bib-0019] de Ruiter, P. C. , A.‐M. Neutel , and J. C. Moore . 1995. “Energetics, Patterns of Interaction Strengths, and Stability in Real Ecosystems.” Science 269: 1257–1260. 10.1126/science.269.5228.1257. 17732112

[men70064-bib-0020] Díaz, S. , J. Kattge , J. H. C. Cornelissen , et al. 2016. “The Global Spectrum of Plant Form and Function.” Nature 529: 167–171. 10.1038/nature16489. 26700811

[men70064-bib-0021] Domonell, A. , M. Brabender , F. Nitsche , M. Bonkowski , and H. Arndt . 2013. “Community Structure of Cultivable Protists in Different Grassland and Forest Soils of Thuringia.” Pedobiologia 56: 1–7. 10.1016/j.pedobi.2012.07.001.

[men70064-bib-0022] Dumack, K. , A. M. Fiore‐Donno , D. Bass , and M. Bonkowski . 2020. “Making Sense of Environmental Sequencing Data: Ecologically Important Functional Traits of the Protistan Groups Cercozoa and Endomyxa (Rhizaria).” Molecular Ecology Resources 20: 398–403. 10.1111/1755-0998.13112. 31677344

[men70064-bib-0023] Dumack, K. , R. Koller , B. Weber , and M. Bonkowsk . 2016. “Estimated Abundance and Diversity of Heterotrophic Protists in South African Biocrusts.” South African Journal of Science 112: 5. 10.17159/sajs.2016/20150302.

[men70064-bib-0024] Dumack, K. , E. Lara , C. Duckert , et al. 2024. “It's Time to Consider the Arcellinida Shell as a Weapon.” European Journal of Protistology 92: 126051. 10.1016/j.ejop.2024.126051. 38194835

[men70064-bib-0025] Dumack, K. , J. Pundt , and M. Bonkowski . 2019. “Food Choice Experiments Indicate Selective Fungivorous Predation in *Fisculla terrestris* (Thecofilosea, Cercozoa).” Journal of Eukaryotic Microbiology 66: 525–527. 10.1111/jeu.12680. 30098099

[men70064-bib-0026] El‐Dib, N. A. 2017. “ *Entamoeba histolytica* : An Overview.” Current Tropical Medicine Reports 4: 11–20. 10.1007/s40475-017-0100-z.

[men70064-bib-0027] Estermann, A. H. , J. Teixeira Pereira Bassiaridis , A. Loos , et al. 2023. “Fungivorous Protists in the Rhizosphere of *Arabidopsis thaliana* – Diversity, Functions, and Publicly Available Cultures for Experimental Exploration.” Soil Biology & Biochemistry 187: 109206. 10.1016/j.soilbio.2023.109206.

[men70064-bib-0085] Finlay, B. J. 2002. “Global Dispersal of Free‐Living Microbial Eukaryote Species.” Science 296: 1061–1063. 10.1126/science.1070710. 12004115

[men70064-bib-0028] Fiore‐Donno, A. M. , J. Freudenthal , M. B. Dahl , C. Rixen , T. Urich , and M. Bonkowski . 2024. “Biotic Interactions Explain Seasonal Dynamics of the Alpine Soil Microbiome.” ISME Communications 4: ycae028. 10.1093/ismeco/ycae028. 38500704 PMC10945362

[men70064-bib-0029] Fiore‐Donno, A. M. , T. Richter‐Heitmann , F. Degrune , et al. 2019. “Functional Traits and Spatio‐Temporal Structure of a Major Group of Soil Protists (Rhizaria: Cercozoa) in a Temperate Grassland.” Frontiers in Microbiology 10: 1332. 10.3389/fmicb.2019.01332. 31244819 PMC6579879

[men70064-bib-0030] Fiore‐Donno, A. M. , J. Weinert , T. Wubet , and M. Bonkowski . 2016. “Metacommunity Analysis of Amoeboid Protists in Grassland Soils.” Scientific Reports 6: 19068. 10.1038/srep19068. 26750872 PMC4707496

[men70064-bib-0031] Flues, S. , D. Bass , and M. Bonkowski . 2017. “Grazing of Leaf‐Associated Cercomonads (Protists: Rhizaria: Cercozoa) Structures Bacterial Community Composition and Function.” Environmental Microbiology 19: 3297–3309. 10.1111/1462-2920.13824. 28618206

[men70064-bib-0032] Fournier, B. , E. Lara , V. E. Jassey , and E. A. Mitchell . 2015. “Functional Traits as a New Approach for Interpreting Testate Amoeba Palaeo‐Records in Peatlands and Assessing the Causes and Consequences of Past Changes in Species Composition.” Holocene 25: 1375–1383. 10.1177/0959683615585842.

[men70064-bib-0033] Freudenthal, J. , K. Dumack , S. Schaffer , M. Schlegel , and M. Bonkowski . 2024. “Algae‐Fungi Symbioses and Bacteria‐Fungi Co‐Exclusion Drive Tree Species‐Specific Differences in Canopy Bark Microbiomes.” ISME Journal 18: wrae206. 10.1093/ismejo/wrae206. 39418324 PMC11630260

[men70064-bib-0034] Freudenthal, J. , F. Ju , H. Bürgmann , and K. Dumack . 2022. “Microeukaryotic Gut Parasites in Wastewater Treatment Plants: Diversity, Activity, and Removal.” Microbiome 10: 27. 10.1186/s40168-022-01225-y. 35139924 PMC8827150

[men70064-bib-0035] Gao, Z. , I. Karlsson , S. Geisen , G. Kowalchuk , and A. Jousset . 2019. “Protists: Puppet Masters of the Rhizosphere Microbiome.” Trends in Plant Science 24: 165–176. 10.1016/j.tplants.2018.10.011. 30446306

[men70064-bib-0036] Geisen, S. , A. M. Fiore‐Donno , J. Walochnik , and M. Bonkowski . 2014. “ *Acanthamoeba* Everywhere: High Diversity of *Acanthamoeba* in Soils.” Parasitology Research 113: 3151–3158. 10.1007/s00436-014-3976-8. 24951165

[men70064-bib-0037] Geisen, S. , R. Koller , M. Hünninghaus , K. Dumack , T. Urich , and M. Bonkowski . 2016. “The Soil Food Web Revisited: Diverse and Widespread Mycophagous Soil Protists.” Soil Biology and Biochemistry 94: 10–18. 10.1016/j.soilbio.2015.11.010.

[men70064-bib-0038] Geisen, S. , I. Laros , A. Vizcaíno , M. Bonkowski , and G. A. de Groot . 2015. “Not All Are Free‐Living: High‐Throughput DNA Metabarcoding Reveals a Diverse Community of Protists Parasitizing Soil Metazoa.” Molecular Ecology 24: 4556–4569. 10.1111/mec.13238. 25966360

[men70064-bib-0039] Geisen, S. , E. A. D. Mitchell , S. Adl , et al. 2018. “Soil Protists: A Fertile Frontier in Soil Biology Research.” FEMS Microbiology Reviews 42: 293–323. 10.1093/femsre/fuy006. 29447350

[men70064-bib-0040] Geisen, S. , J. Rosengarten , R. Koller , C. Mulder , T. Urich , and M. Bonkowski . 2015. “Pack Hunting by a Common Soil Amoeba on Nematodes.” Environmental Microbiology 17: 4538–4546. 10.1111/1462-2920.12949. 26079718

[men70064-bib-0041] Giachello, S. , I. Cantera , A. Carteron , et al. 2023. “Toward a Common Set of Functional Traits for Soil Protists.” Soil Biology and Biochemistry 187: 109207. 10.1016/j.soilbio.2023.109207.

[men70064-bib-0042] Glücksman, E. , T. Bell , R. I. Griffiths , and D. Bass . 2010. “Closely Related Protist Strains Have Different Grazing Impacts on Natural Bacterial Communities.” Environmental Microbiology 12: 3105–3113. 10.1111/j.1462-2920.2010.02283.x. 20602629

[men70064-bib-0043] Guillou, L. , D. Bachar , S. Audic , et al. 2012. “The Protist Ribosomal Reference Database (PR2): A Catalog of Unicellular Eukaryote Small Sub‐Unit rRNA Sequences With Curated Taxonomy.” Nucleic Acids Research 41: D597–D604. 10.1093/nar/gks1160. 23193267 PMC3531120

[men70064-bib-0044] Heck, N. , J. Freudenthal , and K. Dumack . 2023. “Microeukaryotic Predators Shape the Wastewater Microbiome.” Water Research 242: 120293. 10.1016/j.watres.2023.120293. 37421865

[men70064-bib-0045] Ing, B. 1994. “Tansley Review No. 62 the Phytosociology of Myxomycetes.” New Phytologist 126: 175–201. 10.1111/j.1469-8137.1994.tb03937.x.

[men70064-bib-0046] Jassey, V. E. J. , S. Shimano , C. Dupuy , M.‐L. Toussaint , and D. Gilbert . 2012. “Characterizing the Feeding Habits of the Testate Amoebae *Hyalosphenia papilio* and *Nebela tincta* Along a Narrow “Fen‐Bog” Gradient Using Digestive Vacuole Content and 13C and 15N Isotopic Analyses.” Protist 163: 451–464. 10.1016/j.protis.2011.07.006. 21840255

[men70064-bib-0047] Jassey, V. E. J. , R. Walcker , P. Kardol , et al. 2022. “Contribution of Soil Algae to the Global Carbon Cycle.” New Phytologist 234: 64–76. 10.1111/nph.17950. 35103312

[men70064-bib-0087] Jauss, R.‐T. , S. Walden , A. M. Fiore‐Donno , et al. 2020. “From Forest Soil to the Canopy: Increased Habitat Diversity Does Not Increase Species Richness of Cercozoa and Oomycota in Tree Canopies.” Frontiers in Microbiology 11: 592189. 10.3389/fmicb.2020.592189. 33414768 PMC7782269

[men70064-bib-0048] Jauss, R.‐T. , S. Walden , A. M. Fiore‐Donno , et al. 2021. “A Parasite's Paradise: Biotrophic Species Prevail Oomycete Community Composition in Tree Canopies.” Frontiers in Forests and Global Change 4: 668895. 10.3389/ffgc.2021.668895.

[men70064-bib-0049] Kang, S. , A. K. Tice , F. W. Spiegel , et al. 2017. “Between a Pod and a Hard Test: The Deep Evolution of Amoebae.” Molecular Biology and Evolution 34: 2258–2270. 10.1093/molbev/msx162. 28505375 PMC5850466

[men70064-bib-0050] Kassambara, A. 2023a. “ggpubr: “ggplot2” Based Publication Ready Plots.” R package version 0.6.0.

[men70064-bib-0051] Kassambara, A. 2023b. “rstatix: Pipe‐Friendly Framework for Basic Statistical Tests.” R package version 0.7.2.

[men70064-bib-0052] Keller, H. W. , S. E. Everhart , and C. M. Kilgore . 2022. “The Myxomycetes: Introduction, Basic Biology, Life Cycles, Genetics, and Reproduction.” In Myxomycetes: Biology, Systematics, Biogeography and Ecology, edited by C. Rojas and S. L. Stephenson , 1–45. Academic Press. 10.1016/B978-0-12-824281-0.00003-8.

[men70064-bib-0053] Köninger, J. , C. Ballabio , P. Panagos , et al. 2023. “Ecosystem Type Drives Soil Eukaryotic Diversity and Composition in Europe.” Global Change Biology 29: 5706–5719. 10.1111/gcb.16871. 37449367

[men70064-bib-0054] Krause, S. , X. Le Roux , P. A. Niklaus , et al. 2014. “Trait‐Based Approaches for Understanding Microbial Biodiversity and Ecosystem Functioning.” Frontiers in Microbiology 5: 251. 10.3389/fmicb.2014.00251. 24904563 PMC4033906

[men70064-bib-0055] Kulishkin, N. S. , A. A. Surkova , Y. S. Mesentsev , and A. V. Smirnov . 2023. “Three Ways to Eat Spaghettis: Amoebae From Two Amoebozoa Lineages Are Able to Feed on Cyanobacteria of the Genus *Oscillatoria* .” Protistology 17: 38–49. 10.21685/1680-0826-2023-17-1-4.

[men70064-bib-0056] Kylin, H. 2001. “Biodiversity in a Slime Mould: Arthropods Associated With *Brefeldia maxima* .” Mycologist 15: 70–73. 10.1016/S0269-915X(01)80084-7.

[men70064-bib-0057] Lamentowicz, M. , K. Kajukało‐Drygalska , P. Kołaczek , V. E. J. Jassey , M. Gąbka , and M. Karpińska‐Kołaczek . 2020. “Testate Amoebae Taxonomy and Trait Diversity Are Coupled Along an Openness and Wetness Gradient in Pine‐Dominated Baltic Bogs.” European Journal of Protistology 73: 125674. 10.1016/j.ejop.2020.125674. 32200296

[men70064-bib-0058] Lavorel, S. , and E. Garnier . 2002. “Predicting Changes in Community Composition and Ecosystem Functioning From Plant Traits: Revisiting the Holy Grail.” Functional Ecology 16: 545–556. 10.1046/j.1365-2435.2002.00664.x.

[men70064-bib-0059] Lennon, J. T. , Z. T. Aanderud , B. K. Lehmkuhl , and D. R. Schoolmaster Jr. 2012. “Mapping the Niche Space of Soil Microorganisms Using Taxonomy and Traits.” Ecology 93: 1867–1879. 10.1890/11-1745.1. 22928415

[men70064-bib-0060] Lentendu, G. , T. Wubet , A. Chatzinotas , C. Wilhelm , F. Buscot , and M. Schlegel . 2014. “Effects of Long‐Term Differential Fertilization on Eukaryotic Microbial Communities in an Arable Soil: A Multiple Barcoding Approach.” Molecular Ecology 23: 3341–3355. 10.1111/mec.12819. 24888892

[men70064-bib-0061] Lisnerová, M. , H. Pecková , and I. Fiala . 2025. “ Neoparamoeba perurans .” Trends in Parasitology 41: 330–331. 10.1016/j.pt.2024.12.012. 39814641

[men70064-bib-0062] Logares, R. , J. Bråte , S. Bertilsson , J. L. Clasen , K. Shalchian‐Tabrizi , and K. Rengefors . 2009. “Infrequent Marine–Freshwater Transitions in the Microbial World.” Trends in Microbiology 17: 414–422. 10.1016/j.tim.2009.05.010. 19726194

[men70064-bib-0063] Louca, S. , L. W. Parfrey , and M. Doebeli . 2016. “Decoupling Function and Taxonomy in the Global Ocean Microbiome.” Science 353: 1272–1277. 10.1126/science.aaf4507. 27634532

[men70064-bib-0064] Louca, S. , M. F. Polz , F. Mazel , et al. 2018. “Function and Functional Redundancy in Microbial Systems.” Nature Ecology & Evolution 2: 936–943. 10.1038/s41559-018-0519-1. 29662222

[men70064-bib-0065] Martiny, J. B. H. , B. J. M. Bohannan , J. H. Brown , et al. 2006. “Microbial Biogeography: Putting Microorganisms on the Map.” Nature Reviews. Microbiology 4: 102–112. 10.1038/nrmicro1341. 16415926

[men70064-bib-0066] Mulder, C. , F. S. Ahrestani , M. Bahn , et al. 2013. “Connecting the Green and Brown Worlds: Allometric and Stoichiometric Predictability of Above‐ and Below‐Ground Networks.” Advances in Ecological Research 49: 69–175. 10.1016/B978-0-12-420002-9.00002-0.

[men70064-bib-0067] Potapov, A. M. 2022. “Multifunctionality of Belowground Food Webs: Resource, Size and Spatial Energy Channels.” Biological Reviews 97: 1691–1711. 10.1111/brv.12857. 35393748

[men70064-bib-0068] R Core Team . 2023. “R: A Language and Environment for Statistical Computing.” R Foundation for Statistical Computing, Vienna.

[men70064-bib-0069] Ruggiero, M. A. , D. P. Gordon , T. M. Orrell , et al. 2015. “A Higher Level Classification of All Living Organisms.” PLoS One 10: e0119248. 10.1371/journal.pone.0119248. 25923521 PMC4418965

[men70064-bib-0070] Seppey, C. V. W. , D. Singer , K. Dumack , et al. 2017. “Distribution Patterns of Soil Microbial Eukaryotes Suggests Widespread Algivory by Phagotrophic Protists as an Alternative Pathway for Nutrient Cycling.” Soil Biology and Biochemistry 112: 68–76. 10.1016/j.soilbio.2017.05.002.

[men70064-bib-0071] Shadwick, J. D. L. , S. L. Stephenson , and F. W. Spiegel . 2009. “Distribution and Ecology of Protostelids in Great Smoky Mountains National Park.” Mycologia 101: 320–328. 10.3852/08-167. 19537205

[men70064-bib-0072] Singer, D. , E. A. D. Mitchell , R. J. Payne , et al. 2019. “Dispersal Limitations and Historical Factors Determine the Biogeography of Specialized Terrestrial Protists.” Molecular Ecology 28: 3089–3100. 10.1111/mec.15117. 31055860

[men70064-bib-0073] Singer, D. , C. V. W. Seppey , G. Lentendu , et al. 2021. “Protist Taxonomic and Functional Diversity in Soil, Freshwater and Marine Ecosystems.” Environment International 146: 106262. 10.1016/j.envint.2020.106262. 33221595

[men70064-bib-0074] Smirnov, A. , and S. Brown . 2004. “Guide to the Methods of Study and Identification of Soil Gymnamoebae.” Protistology 3: 148–190.

[men70064-bib-0075] Suding, K. N. , S. Lavorel , F. S. Chapin Iii , et al. 2008. “Scaling Environmental Change Through the Community‐Level: A Trait‐Based Response‐And‐Effect Framework for Plants.” Global Change Biology 14: 1125–1140. 10.1111/j.1365-2486.2008.01557.x.

[men70064-bib-0076] Tekle, Y. I. , F. Wang , F. C. Wood , O. R. Anderson , and A. Smirnov . 2022. “New Insights on the Evolutionary Relationships Between the Major Lineages of Amoebozoa.” Scientific Reports 12: 11173. 10.1038/s41598-022-15372-7. 35778543 PMC9249873

[men70064-bib-0077] Tice, A. K. , L. L. Shadwick , A. M. Fiore‐Donno , et al. 2016. “Expansion of the Molecular and Morphological Diversity of Acanthamoebidae (Centramoebida, Amoebozoa) and Identification of a Novel Life Cycle Type Within the Group.” Biology Direct 11: 69. 10.1186/s13062-016-0171-0. 28031045 PMC5192571

[men70064-bib-0078] Urich, T. , A. Lanzén , J. Qi , D. H. Huson , C. Schleper , and S. C. Schuster . 2008. “Simultaneous Assessment of Soil Microbial Community Structure and Function Through Analysis of the Meta‐Transcriptome.” PLoS One 3: e2527. 10.1371/journal.pone.0002527. 18575584 PMC2424134

[men70064-bib-0079] Violle, C. , M.‐L. Navas , D. Vile , et al. 2007. “Let the Concept of Trait Be Functional!” Oikos 116: 882–892. 10.1111/j.0030-1299.2007.15559.x.

[men70064-bib-0080] Visvesvara, G. S. , H. Moura , and F. L. Schuster . 2007. “Pathogenic and Opportunistic Free‐Living Amoebae: *Acanthamoeba* spp., *Balamuthia mandrillaris*, *Naegleria fowleri*, and *Sappinia diploidea* .” FEMS Immunology and Medical Microbiology 50: 1–26. 10.1111/j.1574-695X.2007.00232.x. 17428307

[men70064-bib-0081] Voss, C. , A. M. Fiore‐Donno , M. A. Guerreiro , D. Peršoh , and M. Bonkowski . 2019. “Metatranscriptomics Reveals Unsuspected Protistan Diversity in Leaf Litter Across Temperate Beech Forests, With Amoebozoa the Dominating Lineage.” FEMS Microbiology Ecology 95: fiz142. 10.1093/femsec/fiz142. 31557276

[men70064-bib-0082] Walochnik, J. 2018. “Amoebae.” In Parasitic Protozoa of Farm Animals and Pets, edited by M. Florin‐Christensen and L. Schnittger , 389–412. Springer International Publishing. 10.1007/978-3-319-70132-5_15.

[men70064-bib-0083] Wickham, H. 2011. “ggplot2.” Wiley Interdisciplinary Reviews: Computational Statistics 3: 180–185. 10.1002/wics.147.

[men70064-bib-0084] Woodward, G. , B. Ebenman , M. Emmerson , et al. 2005. “Body Size in Ecological Networks.” Trends in Ecology & Evolution 20: 402–409. 10.1016/j.tree.2005.04.005. 16701403

